# Neural Delays in Processing Speech in Background Noise Minimized after Short-Term Auditory Training

**DOI:** 10.3390/biology13070509

**Published:** 2024-07-08

**Authors:** Erika Skoe, Nina Kraus

**Affiliations:** 1 Department of Speech, Language, and Hearing Sciences, University of Connecticut, Storrs, CT 06269, USA; 2 Department of Communication Sciences, Northwestern University, Evanston, IL 60208, USA; nkraus@northwestern.edu; 3 Cognitive Sciences, Institute for Neuroscience, Northwestern University, Evanston, IL 60208, USA; 4 Department of Neurobiology and Physiology, Northwestern University, Evanston, IL 60208, USA; 5 Department of Linguistics, Northwestern University, Evanston, IL 60208, USA; 6 Department of Otolaryngology, Northwestern University, Evanston, IL 60208, USA

**Keywords:** auditory training, neural plasticity, auditory brainstem response

## Abstract

**Simple Summary:**

Background noise poses a significant detriment to speech perception. Here, as a continuation of our prior work, we provide biological evidence that cognitively-based auditory training lessens the physiologic impact of background noise, resulting in shorter (i.e., earlier) frequency-following response latencies after 20 training sessions.

**Abstract:**

Background noise disrupts the neural processing of sound, resulting in delayed and diminished far-field auditory-evoked responses. In young adults, we previously provided evidence that cognitively based short-term auditory training can ameliorate the impact of background noise on the frequency-following response (FFR), leading to greater neural synchrony to the speech fundamental frequency(F0) in noisy listening conditions. In this same dataset (55 healthy young adults), we now examine whether training-related changes extend to the latency of the FFR, with the prediction of faster neural timing after training. FFRs were measured on two days separated by ~8 weeks. FFRs were elicited by the syllable “da” presented at a signal-to-noise ratio (SNR) of +10 dB SPL relative to a background of multi-talker noise. Half of the participants participated in 20 sessions of computerized training (Listening and Communication Enhancement Program, LACE) between test sessions, while the other half served as Controls. In both groups, half of the participants were non-native speakers of English. In the Control Group, response latencies were unchanged at retest, but for the training group, response latencies were earlier. Findings suggest that auditory training can improve how the adult nervous system responds in noisy listening conditions, as demonstrated by decreased response latencies.

## 1. Introduction

The auditory system is exquisitely tuned to code auditory signals with high temporal precision. This precision is compromised when sound is heard amidst a background of noise. The presence of background noise decreases the number of neurons that fire to the target sound and disrupts neural synchrony, leading to delayed and smaller aggregate neural responses [1,2,3,4,5,6,7,8,9] and reduced speech intelligibility [5,7,10]. 

Our previous work showed that healthy young and older adults can be trained to be better listeners in background noise [11,12]. In young adults [12], we found improved speech perception in noise after 20 sessions of LACE (Listening and Communication Enhancement Program), an adaptive computer-based training program that targets communication in noise [13]. This improved speech perception after ~two months of training was accompanied by more robust frequency-following responses (FFRs) to speech sounds after training, with the training effect being quite specific: results suggested that LACE training affected how the brain responded to speech in background noise without influencing the response under quiet listening conditions. FFRs—phase-locked neural responses to sound recorded at the scalp to periodic or quasi-periodic stimulation [14,15,16]—have been used across a variety of studies as objective neurophysiological indices of short- and long-term auditory neuroplasticity [17,18,19,20]. For the specific FFR stimulus used (“da”) in our previous work, the effect was frequency-specific; training was associated with more robust encoding of lower frequencies (<220 Hz) with no change to the higher frequencies (>220 Hz) of the FFR post-training. These low-frequency spectral enhancements were also most pronounced for the spectrotemporally dynamic (formant-transition) initial portion of the speech stimulus compared to the steady-state later (vowel) portion. Formant transitions and other fast-changing temporal features of speech are perceptually and physiologically less robust to background noise than unchanging acoustic features [21,22,23]. Thus, auditory training appeared to selectively boost the brain’s ability to capture aspects of the speech signal that are most compromised by background noise. Of note is that training-related behavioral improvements emerged for clinical measures of speech perception in noise (SPIN). These perceptual improvements correlated significantly with the magnitude of the FFR to the low-frequencies taken before the onset of training, suggesting that stronger baseline physiologic responses to sound disposes listeners to greater training-related perceptual gains (see also [24]). 

Here, we re-evaluate the training data presented in Song et al. (2012) [12], this time focusing on the latency of the FFR, a dimension not previously analyzed for this dataset. We hypothesized that short-term auditory training that targets speech recognition in noise affects the neurophysiological response to sound by strengthening neural synchrony to the target sound and dampening the response to the background sound, culminating in earlier far-field FFRs to spectrotemporally complex aspects of speech after training and improved perception of speech in background noise. We based this hypothesis on two lines of evidence. First, prolonged auditory training in the form of instrumental musical training is associated with better speech recognition in noise and earlier FFR latencies in background noise [3,6,7]. Second, training-related changes to FFR latency have been observed in an older adult population following 8 weeks of in-home computerized auditory training [11], with similar findings reported for children undergoing speech-sound awareness training [25]. In children [25], training-related changes to the FFR following speech-sound awareness training correlated with improvements in comprehension, suggesting a possible causal relationship between physiological changes and behavioral improvements. 

For young adults, training-related changes to FFR amplitude have been observed across different types of training; however, to our knowledge, none of these reports have evaluated FFR latency before or after training [17,20,26]. We aimed to address this knowledge gap in the current study through a reanalysis of the Song et al., 2012 dataset [12]. One potential explanation for this gap is, that unlike FFR amplitude, which can be measured with automatic routines, FFR latency is often based on a visual inspection of the FFR. Being a phase-locked response, the total duration of the FFR is contingent on the stimulus duration, which, when using speech stimuli, can create dozens of individual peaks that need to be individually measured by a trained rater. As an alternative to manual identification of peaks, we implemented two different, but complementary, approaches to automatically measuring FFR latencies. 

The current analysis adds to the previous work in two specific ways: we focus on the latency of the FFR, and in addition we also provide a more elaborate treatment of the effect of the demographic differences in the dataset. The dataset includes an equal mix of native and non-native English speakers, with an equal representation of each in the Training and Control Groups. In the original analysis, the non-native cohort included anyone who reported having learned English as a second language, regardless of the age they first learned English. Non-native speakers were included in the original sample to broaden the potential range of training outcomes, as difficulties hearing speech in noise tend to be greater for non-native and bilingual speakers. Current theories posit that differences between native and non-native speakers are rooted in linguistic factors and are unlikely to be due to weaknesses in the sensory encoding of sound [27]. Therefore, we predicted that irrespective of native-language status, short-term auditory training would offset the effects of background noise and lead to earlier FFR latencies for the dynamic portion of the speech stimulus (i.e., formant transition). That is, we expected that both native and non-native speakers of English would benefit from training. However, given that non-native speakers had more difficulty perceiving English in noise before training than monolinguals, we predicted that non-native speakers would also have more room to improve, showing potentially greater neurophysiologic changes than monolinguals. Consistent with what has been shown for other aspects of the FFR [28,29], we predicted that latencies would not change for the Control Group over the test-retest interval. We also explored relationships between SPIN and training-related changes to FFR latency, with the underlying assumption of a causal link between speech intelligibility in noise and the degree of neural synchrony in background noise as measured by FFR latency and phase. 

## 2. Materials and Methods

Northwestern University’s Institutional Review Board reviewed and approved all experimental protocols. The basic experimental design and methods for data acquisition are identical to those presented in Song et al., (2012) [12]. We refer the reader to Song et al., 2012 [12] for specific details relating to participant demographics, behavioral testing, and stimulus characteristics. For speech in noise testing, the methods specific to this paper are included in the general methods section. Other methods that pertain only to the present report appear within a separate subheading, “Methods specific to current report”. 

### 2.1. General Methods—Experimental Design and Data Acquisition Methods 

All participants had normal hearing thresholds based on a clinical audiogram, no history of neurological disorders, and minimal to no past musical training. FFRs were collected on two days separated by an average of 8 weeks (referred to hereafter as “test1” and “test2”). Participants were pseudo-randomly assigned to the Trained or Control Groups. Between test1 and test2, the Trained Group underwent 20 sessions of home-based computerized training on LACE. The Control Group did not engage in training or any other prescribed activity between test sessions. In each group, half of the participants were non-native English speakers. A variety of native languages were represented in the sample: Cantonese, Mandarin, Urdu, Panjabi, Farsi, Hebrew, Japanese, Korean, Malayalan, Portuguese, Russian, Azerbaijani, and Serbian-Croatian. Fifteen of the participants were late English learners, operationally defined here as having learned English after the age of 6 years. However, as the Control Group included only five late learners, the effect of late vs. early language learning was not sufficiently powered to undertake.

LACE is a self-paced training program designed for adults with listening difficulties and for people who have recently received a hearing aid. The goal of the training is to improve people’s ability to distinguish voices and understand English that is spoken rapidly or in background noise. Training on these dimensions is accompanied by tasks that target auditory working memory [30]. 

**Figure 1:**
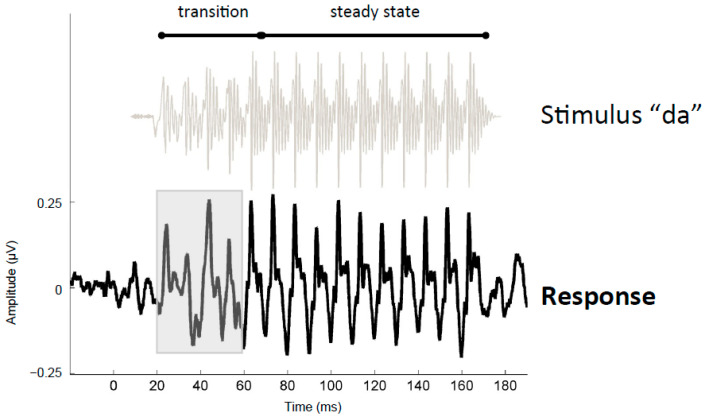
Stimulus and response time-domain waveforms. As a way to illustrate the temporal characteristics and relationships of the “da” stimulus and its frequency-following response (FFR), the grand average response (Trained Group, test1) is plotted below the waveform for the stimulus “da”. The stimulus was presented amidst continuous multi-talker babble (babble not shown). The stimulus waveform was shifted forward by 7.9 ms (to the right) to maximize the visual alignment with the response’s prominent peaks. The speech stimulus was divided acoustically into a formant transition region, during which the speech formants changed linearly as the sound transitioned from the stop burst “d” to the vowel, and a steady-state vocalic region where the formant frequencies were unchanging. The formant transition region had a lower amplitude and more time variation than the steady-state region. The response to the spectrotemporal-dynamic transition is highlighted (20–60 ms).

For each participant, FFRs were recorded to a 170 ms synthesized speech syllable “da”, containing an initial 50 ms formant transition followed by a 120 ms period with stable formants (Figure 1). The stimulus was played to the right ear through insert earphones (Etymōtic Laboratories) at 80 dB SPL with a signal-to-noise ratio (SNR) of +10 dB relative to 6-talker English babble that looped in the background. A quiet condition (with no background noise) was also included in the study design, but it was not included here; our previous findings suggested that the effect of training was nominal for the quiet condition and more evident for the background noise condition. FFRs were differentially recorded using a vertical montage from Cz (active), right earlobe (reference), and forehead (ground) at a 20 kHz sampling rate in Neuroscan Acquire (Compumedics USA, Charlotte, NC, USA). Stimuli were presented in alternating polarity at a rate of 4.3/s. Filtering was performed offline using Neuroscan Edit from 70 to 2000 Hz (Butterworth filter, 12 dB/octave). Responses were epoched over a window spanning 40 ms before and 190 ms after the onset of the stimulus. After excluding trials containing myogenic artifacts, 3000 artifact-free responses to each stimulus polarity were averaged and responses to alternating polarities were averaged to produce the final response. The process of averaging responses to alternating polarities helps to eliminate stimulus artifacts and the cochlear microphonic [31,32]. Myogenic artifacts were defined as trials with activity exceeding ±35 µV.

Training-related changes in perception were assessed using the Hearing in Noise Test (HINT), a clinical test of speech perception in noise (Hearing in Noise Test, Natus Medical Inc., Middleton, WI, USA). This test measures the accuracy of sentence recall as the SNR between the target sentence varies relative to speech-shaped noise, with lower scores reflecting better perceptual abilities (i.e., better sentence recall at more adverse SNRs). HINT sentences are low-context and use simple vocabulary that is appropriate for both adult and child listeners. Target sentences were presented in free field from a loudspeaker placed one meter in front of the participant. The noise was presented from a loudspeaker placed in one of three locations: 0 degrees azimuth (“Noise Front”), 90 degrees to the right (“Noise Right”), or 90 degrees to the left (“Noise Left”) of the participant. For each condition, the final score represents the SNR at which 50% of the sentences were correctly verbally repeated. Song et al. [12]. reported only marginal changes for the Noise Left condition after training in comparison to the Noise Right and Noise Front conditions, where statistically significant changes were found. Given that the FFR testing most closely modeled the HINT Front condition, for the purposes of the current report, the primary statistical analysis focuses on the Noise Front condition. But for the sake of transparency and completeness, we present all HINT conditions, with the caveat that the spatially separated conditions may engage binaural mechanisms not captured in the FFR. Note that two of the Control participants did not complete HINT testing at test2.

QuickSIN, another test of English speech perception in noise (Etymotic Resesarch, Inc., Elk Grove Village, IL, USA), was also administered as part of the original test battery. Performance on the two tests showed a high degree of overlap (r = 0.8, *p* < 0.001), prompting us to focus our primary analysis on HINT. We focused on HINT as the training effects reported in Song et al., 2012 [12] were larger, albeit marginally so. The HINT sentences are also simpler than QuickSIN sentences, making HINT arguably more suitable for non-native speakers. To complete the picture, we also present the results for QuickSIN. 

### 2.2. Methods Specific to Current Report

#### 2.2.1. Participants

Results are reported from 28 Trained (19–35 years, 25.7 ± 4.3 years, 11 males, 16 non-native English speakers) and 27 Control participants (19–31 years, 23.2 ± 3.1 years, 11 males, 15 non-native English speakers). Three Control participants of the original set of 30 reported in Song et al., 2012 [12] were excluded from the current analysis due to low FFR quality (low response SNR) out of concern that the low SNR might compromise latency and phase calculations.

#### 2.2.2. Data Exclusion Criteria

Given the fine-grained timescale over which peak latency measurements are made, latency analyses can be more confounded by signal quality (i.e., low SNR of the response) than the spectral analyses reported in Song et al., 2012 [12]. We assessed signal quality by finding the root-mean-square (RMS) amplitude over the entire response (from 0 to 180 ms) and dividing this value by the RMS of the time period preceding the stimulus (from −40 to 0 ms). This method produces a ratio of ≤1 if there is a complete lack of response (i.e., the evoked response is no bigger than the background noise). Values higher than 1 indicate that the evoked response is larger than any and all ambient noise (electrical, muscular, non-evoked EEG) common to both the response and non-response regions of the averaged electrophysiological data. Participants were excluded from the original dataset (Song et al., 2012 [12]) if the SNR of their response was less than 1.5 at either test1 or test2 and/or if the response SNR changed by more than 50% from test1 to test2 (*n* = 2). An additional Control participant was excluded because s/he was an outlier on the phase measurements (see below), falling >4 standard deviations outside the mean. The SNR of the FFR was calculated by finding the RMS amplitude over the entire response (from 0 to 180 ms) and dividing this value by the RMS of the time period of the recording window that preceded the stimulus (−40 to 0 ms). SNRs did not differ between the Trained and Control Groups at test1 (t(53) = 1.45, *p* = 0.15). 

#### 2.2.3. Measuring Changes in FFR Latency

Training-related changes in FFR latency were assessed in two complementary ways: (1) by evaluating the timing of individual peaks in the response and (2) using a phase-based methodology, the cross-phaseogram [33]. Analyses focused on the frequency-following response (FFR) [14,16] to two time regions: the formant transition (20–60 ms) and the steady-state vowel (60–180 ms). 

##### Peak Latency

**Figure 2:**
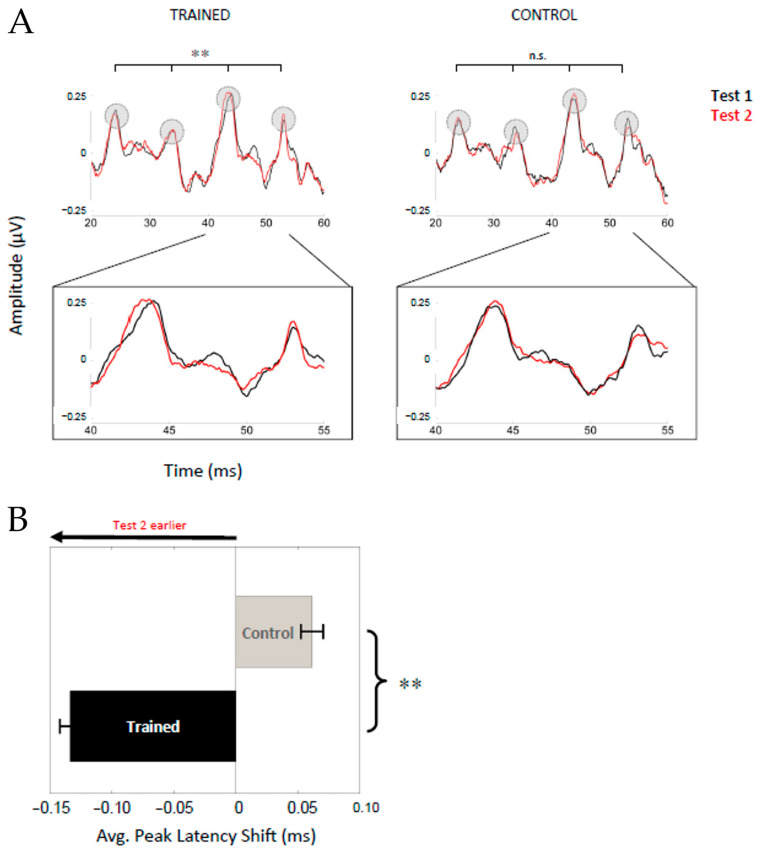
After auditory training, peak latencies are earlier in the neural response to the formant transition. (**A**) The speech-evoked frequency-following response (FFR) includes four prominent positive peaks during the formant transition period at ~24, 34, 44, and 54 ms. The group average time-domain waveforms are plotted for test1 (black) and test2 (red) for the Trained (**left**) and Control Groups (**right**), with the four prominent peaks encircled. To visualize changes in peak timing more clearly, the waveforms are zoomed in to highlight the 40–55 ms window to show the third and fourth major peak in the transition. (**B**) The average peak latency shift is plotted for the Trained (black) and Control (gray) Groups, with error bars representing ±1 standard error of the mean of the four transition peaks. The response was earlier at test2 in the Trained Group (black) but not the Control Group (gray) (main effect of group: F(1,53) = 9.346, *p* = 0.003). Note that (1) group average waveforms can visually blur latency shifts at the major peaks when participants entered the waveform have different baseline latencies and (2) that phase shifts are visually evident not just at the major peaks but also between the major peaks. **, *p* < 0.01.

The FFR to the formant transition included four large peaks, separated by a ~10 ms interval that occurred around 24, 34, 44, and 54 ms (Figure 1 and Figure 2). This series of prominent positive-going peaks continued throughout the steady-state vowel (64, 74…164 ms). These prominent peaks reflect neural synchrony to the periodic aspects of the stimulus, and they occur at roughly the same period as the fundamental frequency F0 of the stimulus (100 Hz). However, the exact latency of each peak was determined not just by the low-frequency F0 but also by higher frequencies in the stimulus, as well as the degree to which different spectral components of the response were degraded by noise [33,34,35]. 

The latency of each prominent peak (15 total) was measured for each participant at test1 and test2 using an automated peak-detection algorithm that identified the maximum amplitude within a 2 ms window (23–25, 33–35, 43–45, 53–55, …163–165 ms) and outputted the corresponding latency. The algorithm was implemented in the MATLAB computing environment (Mathworks, Inc., Natick, MA, USA). The windows were selected based on previous reports using the same stimulus [7,36]. For each peak, the change in latency from test1 to test2 was computed. For each participant, the average latency shift in the response to the formant transition and steady-state region were calculated separately and then each subjected to statistical analysis.

##### Response Phase

The cross-phaseogram method [33] was used to directly compare FFR latency from test1 to test2. The cross-phaseogram is an objective measure of neural timing that compares the temporal synchrony between two signals. This is done by decomposing each signal into its component frequencies and quantifying the phase shift at each frequency. The method was designed on the premise that latency shifts relate to phase shifts in the phase-locked response [33,34,35]. The cross-phase analysis, which was developed to supplement conventional peak-picking methods that extract the latency of individual peaks in the response, is performed on small temporal windows of the response (40-ms in our case), allowing timing shifts to be captured as both a function of frequency and time. A comparison between peak latency and the cross-phaseogram approach in a previous publication showed that the two methods are statistically correlated and thus likely tap in to overlapping (although not necessarily completely identical) neural phenomena [35]. 

**Figure 3:**
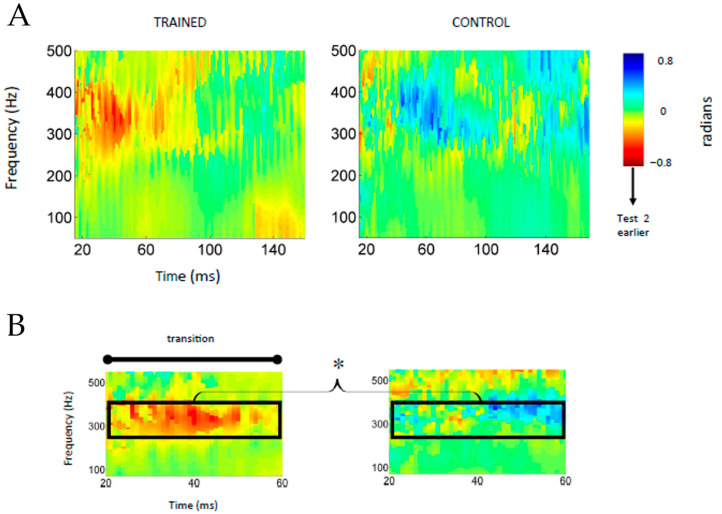
Group average cross-phaseograms for Trained (**left**) and Control (**right**) Groups. Training-related changes in FFR latency were revealed using the cross-phaseogram method, which calculates the phase shift from test1 to test2 as a function of time and frequency. In the cross-phaseogram plot, the more saturated the color is, the greater the phase differences from test1 to test2 are, with warm colors indicating that the response was earlier at test2 than test1. The plot appears green when there was no change in phase from test1 to test2. The full cross-phaseogram (15–170 ms, 70–500 Hz) plotted in panel (**A**) is magnified in panel (**B**) to highlight the training-related phase shifts occurring during the formant transition (20–60 ms) in the 280–420 Hz region. In the Trained Group, the 280–420 Hz is surrounded by a patch of green, suggesting that the phase changes were localized in frequency but extended in time across the transition region. In the Control Group, the average phase shift within this frequency bin did not differ significantly from 0 radians. *, *p* < 0.05.

For the current analysis, cross-phaseograms were generated by calculating cross-power spectral densities in a running-window fashion (40 ms windows) from pairs of responses (i.e., test1 vs. test2) and extracting the phase shifts (in radians) between the two signals at each frequency [33] (Figure 3). To visualize, the cross-phaseogram was then constructed by concatenating the output from each window, creating a three-dimensional image. In the phaseogram, the x-axis represents the midpoint of each time window, and the y-axis represents the frequency (4 Hz resolution). The z-axis, the third dimension, is plotted as a color continuum that reflects the extent of the phase shift between the pair of compared signals. In the colormaps used here, green indicates that the two signals are in phase (0 radians) at a particular time-frequency point. Warm colors (negative radians) indicate that the response at test2 was earlier than test1. 

For analysis purposes, the cross-phaseogram was binned by time and frequency. For each participant, test1-to-test2 phase shifts were averaged over a low frequency (80–220 Hz) and a higher frequency (280–420 Hz) range for the formant transition and steady-state vowel periods. The lower range was selected because for this dataset Song et al., 2012 [12] reported that the spectral power of the FFR was more intense in the 80–220 Hz range after training compared to before, with no changes found for the higher frequency range across test sessions. The 280–420 Hz window was selected because this is where the most prominent phase shifts appear in the Training Group cross-phaseogram (Figure 3), and neural delays that emerge in background noise are greatest near this frequency band [35].

##### Comparing Latency and Phase Shifts

To make comparisons between the two timing measures, phase shifts were converted to latency shifts using the following formula:

L = (D/(360 × f)) × 1000,

where L = change in latency (milliseconds, ms), D = degrees, and f is center frequency. f = 150 Hz for the lower frequency range (80–220 Hz), and f = 350 Hz for the higher frequency range (280–420 Hz). Before applying this formula, radian values were transformed to degrees (1 radian = 57.29 degrees). 

### 2.3. Statistical Analyses

Analyses were performed separately for the response to the formant transition (20–60 ms) and the response to the steady-state vowel (60–180). Test1-to-test2 latency-shifts were compared between the Trained and Control Groups using a mixed-model ANOVA with the Training Group (Trained, Control) and Language Group (native, non-native) as the between-participant factors. For the cross-phase analysis, we performed a mixed-model RMANOVA in which the Frequency Range (2 levels) served as the within-participant factor, and the Training Group (Trained, Control) and Language Group (native, non-native) served as the between participants factors. Bonferroni-corrected post-hoc *t*-tests were performed when appropriate. For each group, one-sample *t*-tests were also computed to determine whether the cross-test latency shifts differed from 0 ms or whether the cross-test phase shifts differed from 0 radians. Changes in response latencies were compared to changes in perceptual abilities. Using Pearson’s correlations, latency and phase measures were compared to performance on HINT-Front.

## 3. Results

### 3.1. Hearing in Noise Test (HINT)

On the HINT-F condition, from test to retest, the Trained Group improved by an average of 1.31 ± 1.93 dB SNR (± values indicate plus and minus one standard deviation), with an initial average performance of −0.58 dB SNR and final performance of −1.88 dB SNR, whereas the Control Group remained comparatively stable (change of −0.04 ± 0.16 dB SNR) (Training Group × Test Interaction: F(1,49) = 7.20, *p* = 0.01, Σ^2^ = 0.01). Not surprisingly, non-native speakers performed more poorly than native speakers overall (F(1,49) = 29.07, *p* < 0.001, Σ^2^ = 0.33); however, the three-way interaction between the Training Group, Language Group, and test session was not significant (F(1,1,49) = 0.18, *p* = 0.176, Σ^2^ = 0.003), suggesting that while non-native speakers had lower performance, native and non-native English speakers benefitted equally from LACE training for this perceptual test. 

For the other SPIN conditions, refer to Table 1 for statistics. Similar trends were seen across the various SPIN tests and conditions. Note that for all SPIN tests, a more negative value indicates better performance, such that background noise had less of an impact on speech perception. If the score improved at re-test, the change had a negative value when comparing the differences between test2 and test1 (change in performance = test2 minus test1). 

### 3.2. Neurophysiology: Formant Transition

#### 3.2.1. Latency Shifts (Figure 2)

The test1-to-test2 latency shift during the transition region was more pronounced for the Trained Group than the Control Group (F(1,53) = 11.47, *p* = 0.001, Σ^2^ = 0.18). For the Control Group, the level of change did not differ from zero ms (0.062 ± 0.244 ms; t(26) = 1.322, *p* = 0.198) (Figure 2). For the Trained Group, the prominent transition response peaks were shifted earlier by an average of 0.13 ± 0.23 ms after training; this degree of shift is statistically different from zero ms (t(27) = −3.066, *p* = 0.005). Similar to the SPIN tests, a more negative latency change indicates a lower impact of background noise at the second test. If the latency is earlier at test2, the change has a negative value when comparing the differences between test2 and test1 (latency shift = test2 − test1). Figure 2 suggests that the effect of training might be focal to certain peaks during the transition (peak~44 ms), prompting a follow-up analysis. When the individual peaks were entered into a mixed-model repeated-measures ANOVA with the Training Group (Control, Trained) as the between-participant factors, and the peak (~24, 34, 44, 54 ms) and test session (session 1, session 2) as within-subject factors, the three-way interaction was not significant (F(1,3,53) = 0.68, *p* = 0.58, Σ^2^ < 0.001). The latency shift for each of the four peaks is given in Table 2. In the Trained Group, non-native speakers showed greater changes, on average (−0.19 ms), than native speakers (−0.10 ms). However, the three-way interaction between the Training Group, Language Group, and time region was not statistically significant (F(1,54) = 3.0, *p* = 0.09, Σ^2 =^ 0.06). 

#### 3.2.2. Phase Shifts

Test1-to-test2 phase shifts were quantified over two frequency regions for the Trained and Control groups. The degree of test-retest phase shift differed between the Trained and Control groups (main effect of Group: F(1,51) = 6.8, *p* = 0.01, Σ^2^ = 0.12), and as a function of frequency (Training Group × Frequency Range interaction: F(1,51) = 5.6 *p* = 0.02, Σ^2^ = 0.09). The effect of training was similar for native and non-native speakers (Training Group × Language Group: F(1,51) =1.5, *p* = 0.28, Σ^2^ = 0.028; Training Group × Language Group × Frequency Range: F(1,1,51) = 3.314, *p* = 0.08, Σ^2^ = 0.06). Post-hoc tests explored the significant interaction between Training Group and the Frequency Range. This analysis revealed that the Trained and Control groups differed on the higher frequency range (*p* = 0.026) but not the lower range (*p* = 0.49). In the higher (280–420 Hz) range, the Trained Group shifted earlier by an average of 0.287 ± 0.596 radians, which is statistically different from zero radians (t(27) = −0.28, *p* = 0.02). However, the Control Group FFR was similar across test sessions in this same frequency range. The phase shift from test1 to test2 did not differ from zero radians (t(26) = 0.95, *p* = 0.35) (Figure 3). For ~68% (19/28) of the Trained Group, there was a negative phase shift between sessions in the 280–420 Hz range compared to ~48% (13/27) of the Control Group. When restricting the analysis to only those participants demonstrating a negative phase shift (i.e., earlier timing at retest) (*n* = 32), the phase shifts were still greater in the Trained Group (*n* = 19) compared to the Control Group (*n* = 13) (t(30) = 2.54, *p* = 0.02).

### 3.3. Neurophysiology: Steady-State Vowel Region

Unlike the transition region, the steady-state region was not associated with training-related changes to FFR latency. The latency of the peaks during the steady-state region was unchanged after training (no main effect of training group in this region (F(1,51) = 1.66, *p* = 0.20, Σ^2^ = 0.01) nor was there a significant interaction between Training Group and Language Group (F(1,51) = 1.50, *p* = 0.23, Σ^2^ = 0.03). For the phase shifts, there was a general trend for the Trained Group shift more at retest (F(1,51) = 3.68, *p* = 0.06, Σ^2^ = 0.07), but there was no Training Group x Frequency Range interaction (F(1,1) = 0.004, *p* = 0.95) (Figure 3). For both peak latency and phase measures, the training effect did not differ between the native and non-native speakers (peak latency: F(1,51) = 0.15, *p* = 0.23, Σ^2^ = 0.03; phase shifts: F(1,51) = 0.49, *p* = 0.487 Σ^2^ = 0.01, respectively). 

### 3.4. Comparing Latency and Phase Shifts

**Figure 4:**
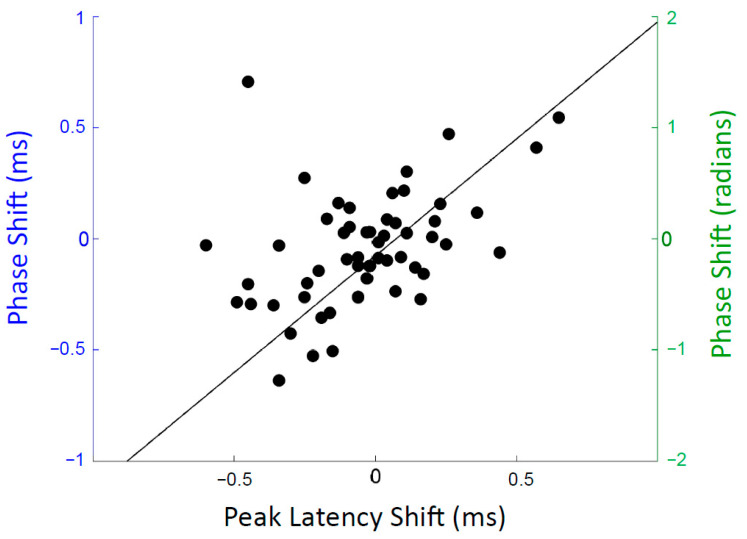
Scatter plot displaying the relationship between latency shifts and phase shifts during the response to the formant transition. To generate this figure, the between-test latency shifts were averaged across the four transition peaks and then correlated with the between-test phase shift in the 280–420 Hz region (r = 0.44, *p* < 0.001). For the phase shifts, the values are reported in milliseconds on the left-hand axis (blue) and in radians on the right-hand axis (green). All participants (*n* = 55) are represented in this figure. Negative radian and ms values indicate earlier timing at test2.

To make direct comparisons between the peak-based latency shifts and frequency-based phase shifts, phase shifts were first converted from radians to milliseconds, as detailed in the Methods section. In the trained group, the average training-related phase shift in the 280–420 Hz range translates to a latency shift of 0.13 ± 0.28 ms, on par with the average peak latency shift of 0.13 ± 0.23 ms, reported above. Thus, similar findings are seen for these two different approaches to measuring temporal changes in the FFR: one which assesses timing based on the latency of discrete peaks, and the other which derives timing shifts from a more continuous, running-window style of analysis. Indeed, the two methods were found to be statistically correlated (r = 0.44, *p* < 0.001, combined dataset of trained and control participants) (Figure 4). However, as can be seen in Figure 4, one (trained) participant stands out, with their phase-shift and latency-shift measurements going in opposite directions. 

### 3.5. Comparisons with Previous Report

In our previous report of this study, which focused on a spectral analysis of the FFR, we reported that the low-frequency phase-locked response increased in spectral magnitude after training during the transition region but higher frequency response was unchanged. [12]. This was confirmed here for a slightly smaller dataset as well (Test Session × Group mixed-model Repeated Measured Analysis of Variance (RMANOVA) for 80–220 Hz: F(1,51) = 5.85, *p* = 0.02, Σ^2^ = 0.10; 280–420 Hz: F(1,51) = 0.12, *p* = 0.73, Σ^2^ = 0.01). The present report shows phase changes associated with training, which were specific to higher frequencies but not lower frequencies components of the FFR. This raises the question of whether spectral response magnitude and timing changes reflect independent phenomena. We explored this possibility: the low-frequency spectral magnitude change and phase change in the high-frequency region do correlate (r = 0.37, *p* <0.05); however, the correlation between magnitude change and peak latency change was not as strong (r = 0.23, *p* = 0.09). 

### 3.6. Correlations with Behavior

**Figure 5:**
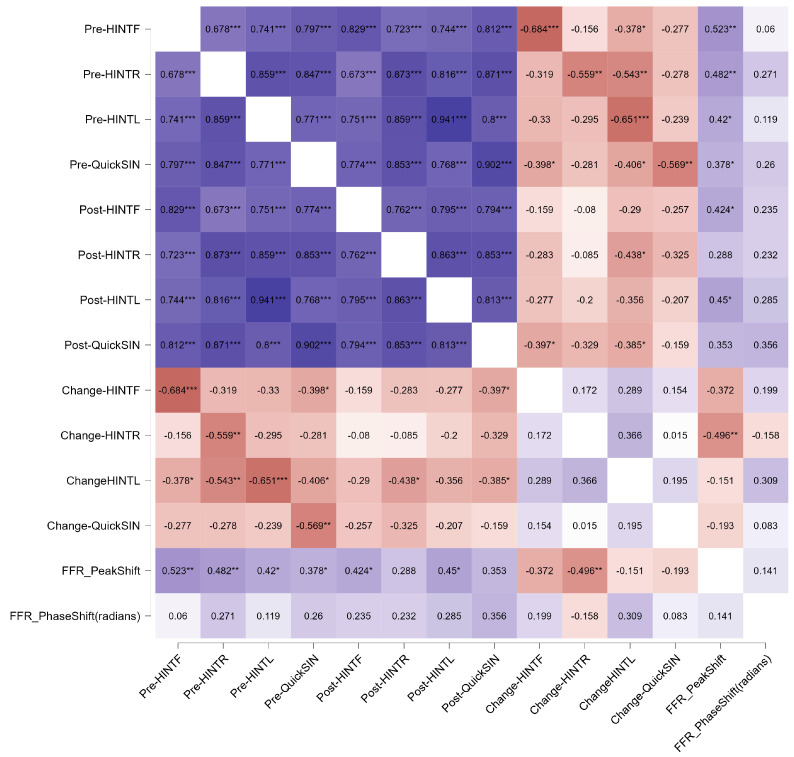
Pearson’s r-heatmap for the Trained Group showing the relationships between performance on the speech perception in noise tests (HINT-F, HINT-R, HINT-L, QuickSIN) between pre- and post-training. Relationships between the change in behavioral performance and change in FFR peak latency and phase are also presented. Positive r-values are coded in blue, and negative r-values are coded in red, with the color gradient reflecting the strength of the correlation. The strongest behavioral performers at pre-test remained the strongest at post-test and showed the smallest behavioral changes but the biggest physiological changes. * *p* < 0.05, ** *p* < 0.01, *** *p* < 0.001.

Next, we considered whether FFR latency changes relate to behavioral outcomes on the SPIN tests both before and after training. For this analysis, we focused only on the Trained Group. Refer to Figure 5 for a heatmap summary (note that the *p*-values are not corrected for multiple comparisons). 

When the degree of latency shift was compared to the amount of behavioral shift for the three HINT conditions and the QuickSIN test, there was a general trend for greater peak shifts to be associated with smaller behavioral changes, with the relationship between the peak latency shift being statistically significant for the HINT-R condition (r = −0.496, *p* < 0.01) and being close to the statistical threshold for the HINT-F condition (r = −0.496, *p* < 0.051). The same general trends were not evident for the phase-based metric (bottom row of the Figure 5 heatmap). 

As presented previously by Song and colleagues [12], participants with the best SPIN scores before training had the smallest behavioral changes with training; conversely, those with the lowest SPIN scores before training had the greatest behavioral changes with training. That is, the direction of the relationship between test1 SPIN scores and the training-related change in SPIN was negative for all tests and conditions (HINT-F, r = −0.684, *p* < 0.001; refer to Table 2 and [12]).

**Figure 6:**
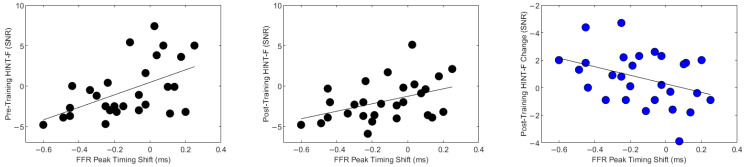
Brain-behavioral relationships. (**left**) Better performance on the Hearing in Noise Test (HINT-F) before training (lower value) was associated with bigger training-related changes in neurophysiology, such that the earlier (lower value) the frequency-following response peaks shifted with training, the better the speech recognition threshold was before training. (**middle**) Similarly, better performance on the Hearing in Noise Test (HINT-F) after training (lower value) was associated with bigger training-related changes in neurophysiology (lower value), such that the earlier the frequency-following response peaks shifted with training, the better the speech recognition threshold was after training. The y-axis shows the HINT score, which represents the dB SNR at which 50% of the sentences were repeated correctly. For HINT, negative scores indicate better performance. (**right**) Smaller behavioral changes with training were associated with bigger training-related changes in neurophysiology. Refer to Figure 5 for r and *p*-values.

Looking specifically at the pre-training SPIN data, those with the best perceptual scores before training tended to have the greatest FFR peak latency shifts after training. Because participants with higher perceptual baselines (i.e., better SPIN scores before training) also had higher SPIN scores after training, this helps to explain why there was a statistically significant relationship between better SPIN scores after training and earlier FFR peak latency shifts (Figure 6). 

## 4. Discussion

Background noise poses a significant detriment to speech perception. But, like other perceptual skills, the ability to detect and understand speech in noise can be improved through training [11,18,24,37]. Here, as a continuation of our prior work [12], we provide biological evidence that LACE training lessens the physiologic impact of background noise, resulting in shorter (i.e., earlier) FFR latencies after 20 training sessions. This finding aligns with what was found for older adults undergoing cognitive-based auditory training [11]. Together, this suggests that the effects are not specific to LACE and that other auditory training programs that take a cognitive approach to improving hearing in noise could yield similar results for young adults. While non-native English speakers had more difficulty hearing spoken English in background noise, they showed similar levels of behavioral and neurophysiological change compared to monolingual English speakers. Consequently, LACE provides a viable training option for native and non-native speakers.

The training-related changes to the FFR latency were focal in nature: changes to FFR latency were limited to the response’s formant-transition region and did not extend into the vowel portion. This is not particularly surprising, as the effects of background noise are generally more pronounced for time-varying than steady-state stimuli [7,38]. Also, for the stimulus used in the current study, the effects of short-term and long-term auditory training appeared specific to the formant transition. For example, for a long-term activity like musical training, earlier FFR latencies have been found for musicians compared to non-musicians for the formant transition but not the steady-state response for this stimulus [3,6,7,39]. Unsurprising given the relatively abbreviated nature of the LACE training, the extent of the latency change observed in the current study was smaller, on average, than the latency differences separating young adult musicians and non-musicians (~0.13 ms vs. ~0.5 ms). However, participants in our study also did not show a uniform level of latency change. While, on average, smaller changes were observed in comparison to long-term training, we found a range of shifts across our trained group, with some showing little to no shift and others showing upwards of a 0.6 ms latency shift. 

### 4.1. Neural Mechanisms

The FFR peaks detected at the scalp likely reflect a confluence of phase-locked activity from multiple auditory structures. When recorded from scalp electrodes, as done here, subcortical sources—especially inferior colliculus—dominate the electrical recordings [40,41,42]. A recent study using techniques to source-localize FFR activity from a 64-channel electrode array found that the subcortical component of the FFR was a stronger predictor of speech perception in noise abilities than cortical components [43]. If that is the case, the physiological changes we observed after auditory training could have been due to the strengthening of the subcortical circuits involved in speech perception in noise. However, based on other studies, training-related changes to auditory cortical function are also likely [44,45], although these changes may affect aspects of cortical function not necessarily reflected in the FFR. 

We observed changes in latency after training. As FFRs were not recorded throughout the training period, the timeline of physiological changes is unknown. Based on a study of non-native speech sound training that recorded FFRs serially during training, it may be that perceptual changes arise first at higher levels and that lower-level changes to the neural encoding of sound emerge at later, more advanced stages of perceptual learning in a fashion consistent with frameworks such as the Reverse Hierarchy Theory and BEAMS hypothesis [46,47,48]. In line with these frameworks, we found an association between better SPIN and a greater reduction in the physiological impact of noise on the FFR after training. Interestingly, those with the greatest training-related latency shifts had better SPIN performance both before and after training. While those with the greatest latency shifts did benefit from training, the degree of benefit on SPIN (measured as the change in behavioral performance) was less than for those who started with worse SPIN performance. This supports the idea that behavioral and physiological changes occur on different timelines and that a listener must progress to a sufficiently advanced level of SPIN ability before substantial physiological changes to FFR latency occur. These physiological changes may help “lock in” the real-world benefits of training. 

The corticofugal pathway is likely essential in guiding top-down mediated changes to sensory coding. The corticofugal pathway is a descending pathway connecting the cortex to subcortical auditory centers. Through this pathway, the cortex can dynamically mediate how subcortical areas respond to sound to produce both excitatory and inhibitory effects [49]. In addition to other roles, the corticofugal pathway is thought to initiate subcortical anti-masking functions that facilitate speech-in-noise perception [50,51] and facilitate the perceptual and attentional foregrounding of sound [49]. This vast efferent pathway, which itself is malleable with training [24,52], has also been argued to play an essential role in driving short-term and long-term subcortical plasticity to behaviorally relevant signals [24,51,53]. 

In the case of our study, auditory training may have heightened attention to speech sounds, particularly low-intensity formant transitions easily obscured by noise. Although the findings are not ubiquitous across the literature [54], a body of literature supports that the FFR is sensitive to the attentional state [54,55,56]. The neurophysiologic recordings were made under passive conditions while the participant watched a movie. Although the attentional state was not monitored, we cannot rule out the possibility that the trained participants were paying more attention to the sounds in the second test session compared to the first. Thus, it is possible that auditory-based cognitive training likely strengthened the top-down mechanisms that counteract perceptual difficulties in noise and improve auditory attention to the target signal, leading to a potentiated response to the target signal and reduced responsiveness to the background noise. An alternative explanation is that training may have strengthened global attentional processes, and this strengthening of efferent processes eventually leads to a default change in (afferent) sound processing without necessarily engaging (or even requiring) conscious attention to the sound stimulus [48]. As a result, after training, the auditory system may respond to the speech syllable as if it were being presented at a higher intensity or a more favorable (i.e., higher) SNR than it actually was. Given the known relationship between FFR latency and intensity, this explanation could account for shorter latencies after training, where incremental increases in intensity produce incremental decreases in latency [57]. In addition to modifying the proportion of the neurons responding to the signal vs. the noise, training may have also increased neural synchrony to the target sound and decreased neuronal jitter across trials, all of which could culminate in earlier far-field responses. Thus, the decrease in latency that was observed after training could reflect various neural mechanisms, including more neurons responding to the target stimulus, fewer neurons responding to the background noise, greater neural synchrony, and/or reduced inter-trial jitter, with the driving force behind these changes presumed to be top-down in nature. 

### 4.2. Clinical Applicability of Our Methodology

Training-induced changes in response timing were observed using two automated methods, one measuring the latency of prominent peaks within pre-defined regions of interest and the other calculating between-session phase shifts over specific time-frequency regions. Both methods minimize the human error and workload associated with manual peak timing measurements, making them clinically tractable. Because of their objectivity, our methods facilitate FFR latency measurements under conditions where manual identification of peaks is difficult, such as when responses are collected in background noise, with limited trials, and/or to long-duration stimuli. 

For future applications, the methods presented here could potentially be streamlined to one metric, given the close correspondence between the two timing metrics. The cross-phaseogram technique has several advantages, especially when recording FFRs to speech and other complex and long-duration sounds. Unlike a click stimulus, which produces a limited number of response peaks, long-duration stimuli produce more. The phaseogram approach eliminates needing to determine a priori which peaks to focus on. The phase method also allows frequency-specific latency shifts to be isolated. Instead of manually identifying the peaks in two recordings, the phase-based method uses a running-window approach to compare the phase of two responses over short intervals. This phase comparison is performed in the spectral domain, yielding frequency-specific phase changes. Decomposing the response in this way has the potential to uncover temporal phenomena that may be imperceptible to the naked eye [35]. 

The phase method has been previously applied to validate test-retest reliability of the brainstem response to complex sounds [58], to document the effect of background noise on the FFR [35], and to examine FFRs to contrastive speech syllables in an animal model [59], as well as in impaired and expert human populations [33,60,61]. Using an existing dataset, we have extended its use to objectively measure auditory training-related changes in healthy adults. Extending this work to an independent (new) dataset would be important for validating and replicating the findings. Although training was found to affect both FFR amplitude and latency, the two indices were not correlated. However, it is currently not clear whether changes in amplitude are a condition for changes in latency; future work hopes to investigate this in a new, independent dataset. 

Another obvious extension of this work would be to use the same phase technique to study the physiological impact of auditory training in clinical populations. There is a growing body of literature assessing the benefits of auditory training in clinical populations, including populations with hearing impairment or neurologic dysfunction [30,62,63,64,65,66]. On the whole, these studies support the idea that auditory training strengthens auditory and cognitive circuits important for hearing in noise, although few studies have adopted randomized control designs [30,62]. This body of work on clinical populations has also largely focused on behavioral indices of auditory plasticity, with a smaller set of studies incorporating physiological metrics (FFRs and auditory evoked cortical potentials) [65,66]. The objective nature of the phase technique makes it a natural complement to existing approaches to studying FFRs and cortical potentials. 

## 5. Conclusions

Communicating in noisy environments is universally tricky, regardless of age and hearing status [67], so even normal-hearing adults can profit from communication-enhancing auditory training. As evidence of this, through a re-analysis of a published dataset, we have shown that short-term auditory training can minimize the influence of background noise on FFR latency, leading to earlier latencies (on average) after training. In addition to further validating the use of short-term auditory training as a means for improving the neural and behavioral mechanisms that support successful auditory perception and communication [28,68,69], our findings reinforce the use of speech-evoked FFRs in the objective assessment of auditory remediation [14,29]. 

## Figures and Tables

**Table 1:** Descriptive statistics for the clinical measures of speech perception in noise taken at each test session. Means, standard deviations (SD), and standard errors (SE) are presented separately for the non-native and native English speakers (native = 0 and 1, respectively) for the Control and Trained groups (Trained = 0 and 1, respectively). The main effects and interactions from the repeated measures ANOVA are presented at the bottom.

HINT-F			HINT-F (SNR)			HINT-R (SNR)			HINT-L (SNR)			QUICKSIN (SNR Loss)		
Session	Trained	Native	Mean	SD	SE	Mean	SD	SE	Mean	SD	SE	Mean	SD	SE
**1**	0	0	0.814	2.712	0.725	−4.517	2.865	0.74	−3.813	3.353	0.866	3.528	3.567	0.921
		1	−3.055	0.662	0.2	−8.8	1.76	0.531	−8.473	1.054	0.318	−0.382	0.83	0.25
	1	0	0.839	3.486	0.822	−4	3.751	0.884	−4.241	4.649	1.096	4.008	3.742	0.882
		1	−3.13	0.757	0.239	−7.77	2.063	0.653	−7.87	1.025	0.324	−0.592	0.976	0.309
**2**	0	0	0.564	2.119	0.566	−4.8	2.745	0.709	−3.487	2.898	0.748	2.878	2.843	0.734
		1	−2.645	0.753	0.227	−8.109	1.768	0.533	−8.682	1.064	0.321	−0.771	0.853	0.257
	1	0	−1.117	2.627	0.619	−5.606	3.174	0.748	−4.889	3.652	0.861	2.303	2.884	0.68
		1	−3.272	1.617	0.511	−8.97	1.122	0.355	−8.23	0.821	0.26	−1.901	1.064	0.336
			*Statistics*			*Statistics*			*Statistics*			*Statistics*		
*Trained × Test Session*			*F(1,49) = 7.20, p = 0.01, Σ^2^ = 0.01*			*F(1,49) = 12.58, p < 0.001, Σ^2^ = 0.02*			*F(1,49) = 1.10, p = 0.298, Σ^2^ <0.001*			*F(1,50) = 1.77 p = 0.19, Σ^2^ = 0.001*		
*Trained × Test Session × Native*			*F(1,1,49) = 0.18, p = 0.176, Σ^2^ = 0.003*			*F(1,1,49) = 0.40, p = 0.53, Σ^2^ < 0.001*			*F(1,1,49) = 0.95, p = 0.335, Σ^2^ < 0.001*			*F(1,1,50) = 0.015, p = 0.902, Σ^2^ < 0.001*		
*Native*			*F(1,49) = 29.07, p < 0.001, Σ^2^ = 0.33*			*F(1,49) = 25.57, p < 0.001, Σ^2^ = 0.31*			*F(1,49) = 25.6, p < 0.001, Σ^2^ = 0.33*			*F(1,50) = 35.2, p < 0.001, Σ^2^ = 0.36*		

**Table 2:** Descriptive statistics of the latency change (in milliseconds, ms) for each of the four transition peaks for the Control (Trained = 0) and Trained (Trained = 1) groups. SD = standard deviation; SE = standard error. Negative values indicate that the peak shifted earlier on average at test session 2. The peaks occurred at approximately ~24, 34, 44, and 54 ms.

Peak	Trained	Mean (Change in ms)	SD	SE
**1**	0	0.015	0.611	0.118
	1	−0.02	0.529	0.1
2	0	0.106	0.586	0.113
	1	−0.184	0.63	0.119
3	0	0.041	0.465	0.09
	1	−0.107	0.347	0.066
4	0	0.087	0.659	0.127
	1	−0.223	0.574	0.108

## Data Availability

Data are available upon request from the authors.

## References

[1] Sachs M.B., Voigt H.F., Young E.D. (1983). Auditory nerve representation of vowels in background noise. J. Neurophysiol..

[2] Simmons A.M., Schwartz J.J., Ferragamo M. (1992). Auditory nerve representation of a complex communication sound in background noise. J. Acoust. Soc. Am..

[3] Strait D.L., Parbery-Clark A., Hittner E., Kraus N. (2012). Musical training during early childhood enhances the neural encoding of speech in noise. Brain Lang..

[4] Li X., Jeng F.C. (2011). Noise tolerance in human frequency-following responses to voice pitch. J. Acoust. Soc. Am..

[5] Anderson S., Skoe E., Chandrasekaran B., Kraus N. (2010). Neural timing is linked to speech perception in noise. J. Neurosci..

[6] Parbery-Clark A., Anderson S., Hittner E., Kraus N. (2012). Musical experience offsets age-related delays in neural timing. Neurobiol. Aging.

[7] Parbery-Clark A., Skoe E., Kraus N. (2009). Musical experience limits the degradative effects of background noise on the neural processing of sound. J. Neurosci..

[8] Delgutte B., Kiang N.Y. (1984). Speech coding in the auditory nerve: V. Vowels in background noise. J. Acoust. Soc. Am..

[9] Prevost F., Laroche M., Marcoux A.M., Dajani H.R. (2012). Objective measurement of physiological signal-to-noise gain in the brainstem response to a synthetic vowel. Clin. Neurophysiol..

[10] Song J.H., Skoe E., Banai K., Kraus N. (2011). Perception of speech in noise: Neural correlates. J. Cogn. Neurosci..

[11] Anderson S., White-Schwoch T., Parbery-Clark A., Kraus N. (2013). Reversal of age-related neural timing delays with training. Proc. Natl. Acad. Sci. USA.

[12] Song J.H., Skoe E., Banai K., Kraus N. (2012). Training to improve hearing speech in noise: Biological mechanisms. Cereb. Cortex.

[13] Sweetow R.W., Sabes J.H. (2006). The need for and development of an adaptive Listening and Communication Enhancement (LACE) Program. J. Am. Acad. Audiol..

[14] Skoe E., Kraus N. (2010). Auditory brain stem response to complex sounds: A tutorial. Ear Hear..

[15] Krizman J., Kraus N. (2019). Analyzing the FFR: A tutorial for decoding the richness of auditory function. Hear. Res..

[16] Moushegian G., Rupert A.L., Stillman R.D. (1973). Laboratory note. Scalp-recorded early responses in man to frequencies in the speech range. Electroencephalogr. Clin. Neurophysiol..

[17] Carcagno S., Plack C.J. (2011). Subcortical plasticity following perceptual learning in a pitch discrimination task. J. Assoc. Res. Otolaryngol..

[18] Song J.H., Skoe E., Wong P.C., Kraus N. (2008). Plasticity in the adult human auditory brainstem following short-term linguistic training. J. Cogn. Neurosci..

[19] Musacchia G., Sams M., Skoe E., Kraus N. (2007). Musicians have enhanced subcortical auditory and audiovisual processing of speech and music. Proc. Natl. Acad. Sci. USA.

[20] Reetzke R., Xie Z., Llanos F., Chandrasekaran B. (2018). Tracing the trajectory of sensory plasticity across different stages of speech learning in adulthood. Curr. Biol..

[21] Cunningham J., Nicol T., King C., Zecker S.G., Kraus N. (2002). Effects of noise and cue enhancement on neural responses to speech in auditory midbrain, thalamus and cortex. Hear. Res..

[22] Nishi K., Lewis D.E., Hoover B.M., Choi S., Stelmachowicz P.G. (2010). Children’s recognition of American English consonants in noise. J. Acoust. Soc. Am..

[23] Tallal P., Stark R.E. (1981). Speech acoustic-cue discrimination abilities of normally developing and language-impaired children. J. Acoust. Soc. Am..

[24] De Boer J., Thornton A.R. (2008). Neural correlates of perceptual learning in the auditory brainstem: Efferent activity predicts and reflects improvement at a speech-in-noise discrimination task. J. Neurosci..

[25] Russo N.M., Nicol T.G., Zecker S.G., Hayes E.A., Kraus N. (2005). Auditory training improves neural timing in the human brainstem. Behav. Brain Res..

[26] Skoe E., Chandrasekaran B., Spitzer E.R., Wong P.C., Kraus N. (2014). Human brainstem plasticity: The interaction of stimulus probability and auditory learning. Neurobiol. Learn. Mem..

[27] Krizman J., Bradlow A.R., Lam S.S.-Y., Kraus N. (2017). How bilinguals listen in noise: Linguistic and non-linguistic factors. Biling. Lang. Cogn..

[28] Carcagno S., Plack C.J. (2011). Pitch discrimination learning: Specificity for pitch and harmonic resolvability, and electrophysiological correlates. J. Assoc. Res. Otolaryngol..

[29] Hornickel J., Zecker S.G., Bradlow A.R., Kraus N. (2012). Assistive listening devices drive neuroplasticity in children with dyslexia. Proc. Natl. Acad. Sci. USA.

[30] Lawrence B.J., Jayakody D.M., Henshaw H., Ferguson M.A., Eikelboom R.H., Loftus A.M., Friedland P.L. (2018). Auditory and cognitive training for cognition in adults with hearing loss: A systematic review and meta-analysis. Trends Hear..

[31] Aiken S.J., Picton T.W. (2008). Envelope and spectral frequency-following responses to vowel sounds. Hear. Res..

[32] Chimento T.C., Schreiner C.E. (1990). Selectively eliminating cochlear microphonic contamination from the frequency-following response. Electroencephalogr. Clin. Neurophysiol..

[33] Skoe E., Nicol T., Kraus N. (2011). Cross-phaseogram: Objective neural index of speech sound differentiation. J. Neurosci. Methods.

[34] John M.S., Picton T.W. (2000). Human auditory steady-state responses to amplitude-modulated tones: Phase and latency measurements. Hear. Res..

[35] Tierney A., Parbery-Clark A., Skoe E., Kraus N. (2011). Frequency-dependent effects of background noise on subcortical response timing. Hear. Res..

[36] Song J.H., Nicol T., Kraus N. (2011). Test-retest reliability of the speech-evoked auditory brainstem response. Clin. Neurophysiol..

[37] Whitton J.P., Hancock K.E., Shannon J.M., Polley D.B. (2017). Audiomotor perceptual training enhances speech intelligibility in background noise. Curr. Biol..

[38] Anderson S., Chandrasekaran B., Yi H.G., Kraus N. (2010). Cortical-evoked potentials reflect speech-in-noise perception in children. Eur. J. Neurosci..

[39] Parbery-Clark A., Anderson S., Hittner E., Kraus N. (2012). Musical experience strengthens the neural representation of sounds important for communication in middle-aged adults. Front. Aging Neurosci..

[40] Lerud K.D., Hancock R., Skoe E. (2023). A high-density EEG and structural MRI source analysis of the frequency following response to missing fundamental stimuli reveals subcortical and cortical activation to low and high frequency stimuli. NeuroImage.

[41] Bidelman G.M. (2018). Subcortical sources dominate the neuroelectric auditory frequency-following response to speech. NeuroImage.

[42] Coffey E.B., Nicol T., White-Schwoch T., Chandrasekaran B., Krizman J., Skoe E., Zatorre R.J., Kraus N. (2019). Evolving perspectives on the sources of the frequency-following response. Nat. Commun..

[43] Bidelman G.M., Momtaz S. (2021). Subcortical rather than cortical sources of the frequency-following response (FFR) relate to speech-in-noise perception in normal-hearing listeners. Neurosci. Lett..

[44] Hayes E.A., Warrier C.M., Nicol T.G., Zecker S.G., Kraus N. (2003). Neural plasticity following auditory training in children with learning problems. Clin. Neurophysiol..

[45] Pantev C., Wollbrink A., Roberts L.E., Engelien A., Lütkenhöner B. (1999). Short-term plasticity of the human auditory cortex. Brain Res..

[46] Ahissar M., Nahum M., Nelken I., Hochstein S. (2009). Reverse hierarchies and sensory learning. Philos. Trans. R. Soc. Lond. B Biol. Sci..

[47] Nahum M., Nelken I., Ahissar M. (2008). Low-level information and high-level perception: The case of speech in noise. PLoS Biol..

[48] Kraus N. (2021). Memory for sound: The BEAMS hypothesis [Perspective]. Hear. Res..

[49] Usrey W.M., Sherman S.M. (2019). Corticofugal circuits: Communication lines from the cortex to the rest of the brain. J. Comp. Neurol..

[50] Liberman M.C., Guinan J.J. (1998). Feedback control of the auditory periphery: Anti-masking effects of middle ear muscles vs. olivocochlear efferents. J. Commun. Disord..

[51] Suga N., Xiao Z., Ma X., Ji W. (2002). Plasticity and corticofugal modulation for hearing in adult animals. Neuron.

[52] Brashears S.M., Morlet T.G., Berlin C.I., Hood L.J. (2003). Olivocochlear efferent suppression in classical musicians. J. Am. Acad. Audiol..

[53] Bajo V.M., Nodal F.R., Moore D.R., King A.J. (2010). The descending corticocollicular pathway mediates learning-induced auditory plasticity. Nat. Neurosci..

[54] Galbraith G.C., Olfman D.M., Huffman T.M. (2003). Selective attention affects human brain stem frequency-following response. Neuroreport.

[55] Hairston W.D., Letowski T.R., McDowell K. (2013). Task-Related Suppression of the Brainstem Frequency following Response. PLoS ONE.

[56] Hartmann T., Weisz N. (2019). Auditory cortical generators of the Frequency Following Response are modulated by intermodal attention. NeuroImage.

[57] Bidelman G., Powers L. (2018). Response properties of the human frequency-following response (FFR) to speech and non-speech sounds: Level dependence, adaptation and phase-locking limits. Int. J. Audiol..

[58] Hornickel J., Knowles E., Kraus N. (2012). Test-retest consistency of speech-evoked auditory brainstem responses in typically-developing children. Hear. Res..

[59] Warrier C.M., Abrams D.A., Nicol T.G., Kraus N. (2011). Inferior colliculus contributions to phase encoding of stop consonants in an animal model. Hear. Res..

[60] Parbery-Clark A., Tierney A., Strait D.L., Kraus N. (2012). Musicians have fine-tuned neural distinction of speech syllables. Neuroscience.

[61] Neef N.E., Schaadt G., Friederici A.D. (2017). Auditory brainstem responses to stop consonants predict literacy. Clin. Neurophysiol..

[62] Henshaw H., Ferguson M.A. (2013). Efficacy of individual computer-based auditory training for people with hearing loss: A systematic review of the evidence. PLoS ONE.

[63] Fisher M., Holland C., Merzenich M.M., Vinogradov S. (2009). Using neuroplasticity-based auditory training to improve verbal memory in schizophrenia. Am. J. Psychiatry.

[64] Buriti A.K.L., Gil D. (2022). Mild traumatic brain injury: Long-term follow-up of central auditory processing after auditory training. J. Audiol. Otol..

[65] Gaeta L., Stark R.K., Ofili E. (2021). Methodological Considerations for Auditory Training Interventions for Adults With Hearing Loss: A Rapid Review. Am. J. Audiol..

[66] Stropahl M., Besser J., Launer S. (2020). Auditory training supports auditory rehabilitation: A state-of-the-art review. Ear Hear..

[67] Assmann P., Summerfield Q. (2004). The Perception of Speech Under Adverse Conditions. Speech Processing in the Auditory System.

[68] Merzenich M.M., Jenkins W.M., Johnston P., Schreiner C., Miller S.L., Tallal P. (1996). Temporal processing deficits of language-learning impaired children ameliorated by training. Science.

[69] Millward K.E., Hall R.L., Ferguson M.A., Moore D.R. (2011). Training speech-in-noise perception in mainstream school children. Int. J. Pediatr. Otorhinolaryngol..

